# Supplementary material for: Stakeholder acceptance and attitudes toward dengue prevention: A study on fogging and autocidal traps techniques in Malaysia
Source: PLoS One. 2025 Jul 16;20(7):e0323061. doi: 10.1371/journal.pone.0323061 (PMC12266440; doi:10.1371/journal.pone.0323061)
Supplement: Attachment — Submitted filename: Response to Reviewers.pdf [file pone.0323061.s002.pdf]

Dear Editor and reviewers,

Thank you for your suggestion. We have tried to answer almost all the concerns of academic editors and reviewers.

REVIEWER 1

R#1 : In this manuscript I evaluate the proposal for an alternative selective chemical control with proven effectiveness in previous studies and the contribution that complements its feasible possibility in its useful application, with acceptance and possible participation of involved actors, including the population exposed to the problem. The validation of the measurement questionnaire, data collection, analysis and results were prepared with sufficient methodological rigor.

Response:

Thank you for your comment. We appreciate this recognition of our methodological approach and remain open to any further suggestions for strengthening the clarity and impact of our findings.

REVIEWER 2

Reviewer #2: Relevance in the Introduction:

It is important to highlight the importance of health promotion in relation to the increase in patients with dengue in localities where there was previously no presence of autochthonous cases, that is, the increase in endemic localities. In seeking economically and operationally viable solutions, public opinion from different sectors plays a transcendental role in the effectiveness of dengue prevention and control work, that is, intersectoral opinion and participation. Evaluating the acceptance of different control techniques, when perhaps a change in the operational activities of a particular area is intended, is crucial, as is evaluating the parameters in each of the localities, since biotic and abiotic conditions, relief, population density, ambient temperature, annual rainfall, among others, largely determine what type of actions should be implemented in each locality, taking into account its own challenges in the operational work that is desired to be implemented. Lack of knowledge about the disease itself, as well as about the activities implemented in the population, could cause problems or delays in carrying out the activity, due to the fact that a certain number of variables to be implemented are not considered, as well as making the population aware of the new techniques to be implemented. In addition to this, the health culture in each of the regions to be established greatly favors or hinders the impact of one or several strategies. As mentioned, the rapid expansion of the disease, together with the adaptability of the vectors to new environments, that is, environments where the disease was not common, can be associated with the effectiveness of the different activities carried out for prevention and control of dengue. Other factors that largely determine the presence of potential breeding sites are the urban planning of the same areas. It is important to include new innovations in control, as well as the evaluation of acceptance in the population in each of the different regions. Allowing the experimentation of different techniques based on monitoring by ovitraps is essential to improve the initial versions of this methodology, that is, the more new designs

are allowed to be experimented with in the autocidal ovitraps, the more efficiently they will be able to achieve the objective of reducing the density of the vector.

Including the participation of the different institutions, both from the public and private educational sectors, the environmental sector, institutions related to the supply of drinking water, institutions related to the collection of urban solid waste, public and private health institutions, research institutions as well as local and federal governments of each of the nations, added together in a single objective which is the reduction of dengue cases. Likewise, the creation of public policies in each of the local governments could help to pressure for the creation of new strategies that help to mitigate this condition.

Response:

Thank you for your comment. This is the revision and the refined version of the comment:

Dengue fever, a viral infection spread by mosquitoes, continues to be a significant global public health issue, especially in tropical and subtropical regions. It is among the most widespread vector-borne diseases and is rapidly proliferating. The disease is predominantly transmitted by female Aedes aegypti mosquitoes (the principal vector) and Aedes albopictus mosquitoes (the secondary vector), with severe dengue resulting in a mortality rate of up to 20%. The number of dengue cases documented has risen tenfold over the last 20 years, from 505,430 cases in 2000 to over 5.2 million cases in 2019 [4]. Dengue fever is widespread in over 125 countries, impacting almost 3.6 billion individuals globally.

Despite dengue's severity and prevalence, there is currently no readily accessible vaccine or specialized antiviral cure for the illness (WHO, 2024). Thus, vector management continues to be the principal technique for reducing dengue transmission (PAHO, 2024). Conventional preventive techniques, such as chemical fogging, are extensively employed to eradicate infected mosquito populations during epidemics. However, these tactics have proven poor long-term efficiency in preventing dengue transmission, as Aedes mosquitos have evolved pesticide resistance, and fogging alone is insufficient to terminate epidemics [8]. For dengue to keep spreading, only two to three mature female mosquitoes need to hatch every day for every 100 people (Praveen & Saha 2023); as little as two to three adults are needed.

Given the increasing prevalence of dengue cases, it is imperative to implement creative and sustainable vector control techniques. A promising method is the implementation of autocidal gravid ovitraps (AGOs), which have shown sustained efficacy in dengue-endemic regions [5,6]. AGOs are enhanced iterations of standard ovitraps intended to capture and eliminate mosquito larvae and gravid female mosquitoes, hence diminishing vector populations early (García-Rejón 2011). Research done in Puerto Rico and Australia has shown that AGOs can effectively cut down on mosquito numbers by a large amount [5,6]. Nevertheless, there is still little widespread use of autocidal traps in South America, especially in Colombia, one of the most impacted nations in the area [7].

The efficiency of any dengue control plan is determined by scientific efficacy as well as public perception, acceptance, and stakeholder engagement. Studies demonstrate that community involvement markedly improves adherence and fosters the enduring viability of vector control initiatives (PAHO, 2024). The implementation of novel control methods is affected by various factors, such as perceived risks, perceived benefits, faith in public health authorities, and

sociocultural influences (Alobuia, 2015). Also, urban planning is very important for preventing dengue because Aedes aegypti mosquitoes love to breed in places with bad sewage systems, poor waste management, and many individuals (Praveen & Saha 2023).

Comprehending stakeholder perceptions of diverse dengue management strategies is crucial for formulating effective public health communication and engagement activities. Stakeholders, such as public health officials, community leaders, and local citizens, are crucial to the execution and efficacy of dengue preventive initiatives. Their apprehensions, awareness levels, and acceptance substantially influence the efficacy and durability of dengue prevention programs (WHO, 2024).

This study seeks to examine and contrast stakeholder acceptance and perspectives about fogging and autocidal traps as methods of dengue prevention. This research will use SPSS and one-way MANOVA analysis to investigate variances in community views among different stakeholder groups. The results will offer significant insights into each strategy's perceived risks and advantages, subsequently informing the creation of more successful, community-oriented dengue preventive initiatives.

Reviewer #2: Adequate methodology :

The quantitative measurement in the perspectives of attitudes and acceptance of measures for the prevention of dengue is adequate, it could be mentioned that it is also a qualitative research, since it is focused on determining the opinion of the different target populations. It is very important to highlight this agreement of the acceptance of a methodology in both groups, to ensure the population benefits from the new activity to be implemented

Response: Thank you for your insightful commentary. This is the revision and the refined version of the comment:

The relationship between the variables listed in the framework of concepts was investigated in this study using a method based on quantitative research. This inquiry requires stakeholder viewpoints to create an inventory of attitudes and acceptance of dengue prevention and control measures using a survey. The sample size is determined according to Krejcie and Morgan's (1970) recommendation. To get a power value of 0.80 and identify a medium-sized impact (f=0.25) with a significance level of p=0.05 and a margin of error of u=10, a sample size of 25 respondents is required for the target population. As shown in Table 1.1, the sample size of 415 (n=415) is optimal for the study, as it is within the range of 384 to 500 samples. These samples are of people aged 18 and up and have been stratified according to the target category. Although our study employed a quantitative methodology to ensure objective and generalizable findings, we advise that future research use qualitative techniques such as focus groups or interviews to understand better stakeholder perceptions and the motivations, concerns, and contextual factors influencing public acceptance of dengue prevention measures.

Reviewer #2: Discussion :

All activities focused on preventing dengue are important for public health. It is important to mention the average score of the response of the most significant benefit received from nebulization, which gives rise to implementing new control and prevention measures, but it will be the sum of many strategies that determine the optimal impact of the reduction of this

disease. This is focused on the fact that depending on the population at risk in a given area and the environmental variables, the type of methodology to be implemented will be determined. One could mention the type of variables that exist in the Klang Valley in Malaysia: the socioeconomic and environmental variables. Likewise, the lack of knowledge of the disease in many areas can change the entire methodology of a prevention and control activity, as it is mentioned that 38.8% of those surveyed are not sure that the problem can be solved. It is very good that they mention their community's health benefits and quality of life.

Response: Thank you for your valuable feedback. This is the revision and the refined version of the comment:

Dengue fever still remains a considerable global public health concern, necessitating extensive and flexible management efforts to reduce its transmission. Conventional techniques like nebulization (fogging) have been extensively employed to eradicate adult Aedes mosquitoes, yet their efficacy is sometimes transient and significantly influenced by environmental and operational variables (García-Rejón et al., 2011; BMC Public Health, 2024). Although fogging is regarded as advantageous, research demonstrates that it fails to yield sustained decreases in mosquito populations, highlighting the necessity for alternate and complementary control methods (WHO, 2024). The intricacy of dengue transmission underscores the need for multifaceted management measures, as outbreaks are affected by environmental, socioeconomic, and urbanization factors (Praveen & Saha, 2023).

Due to growing urbanization and poor waste management, which provide perfect conditions for mosquito reproduction, Malaysia's Klang Valley region—which accounts for 57% of all dengue infections in the nation—faces additional difficulties (CDC Taiwan, 2015). Investigations into low-income neighbourhoods in Klang Valley highlight how inadequate housing, incorrect water storage, and ineffective drainage systems contribute to increased mosquito populations (Bujang et al., 2023). These findings underscore the necessity of comprehensive dengue preventive efforts that consider both environmental and socioeconomic aspects in conjunction with conventional vector control strategies.

Public perception significantly influences the adoption of dengue prevention strategies, as acceptability is mostly determined by perceived advantages and dangers (Fischhoff et al., 2024). Research from Klang Valley, Malaysia, reveals that participants recognize the advantages of both fogging and autocidal traps in dengue prevention. Scientific evaluations indicate that autocidal traps (mean score: 5.41) are regarded as more effective than fogging (5.26), underscoring the significance of implementing technology-driven interventions (PLOS One, 2024). Therefore, public popularity rises when a technology like an autocidal trap exhibits distinct benefits. The public's readiness to endorse biotechnology applications is chiefly influenced by their assessment of its advantages, whereas perceived risks—such as anticipated financial or health detriments—serve as obstacles to adoption (Fischhoff et al., 2024).

The perceived advantage is a vital factor influencing public support for dengue prevention initiatives. A favorable view enhances adoption, whereas perceived dangers, including economic losses or uncertainty, may diminish public readiness to embrace new control measures (Fischhoff et al., 2024). Respondents in the Klang Valley generally think that autocidal traps and fogging offer substantial advantages in avoiding dengue. Nevertheless, experts contend that the autocidal trap method (5.41) is superior to fogging (5.26) because to the difference in mean

scores achieved. This indicates that definitive proof of enhanced effectiveness will affect public choice, resulting in increased social acceptability of novel dengue prevention methods ([9,10]). Risk perception and benefit assessment are interconnected in the evaluation of dengue prevention strategies. According to Fischhoff et al. (2024), there is a clear correlation between perceived risk and perceived benefit, which means that a higher risk perception may make people less trusting of a new solution. A study in Seremban, Malaysia, indicated that risk perception mediates the association between public attitudes and dengue preventative practices, underscoring the necessity for community education and awareness initiatives ([14,15]).

The concept is endorsed by philosophical traditions asserting that behavioral modification commences with risk awareness, as individuals are more inclined to alter their health practices upon recognizing concrete harm to their well-being ([16]). Moreover, altering one's behavior becomes unavoidable when there is a significant perceived risk of disease, as evidenced by a study in Singapore that documented measures to prevent in groups extremely vulnerable to dengue transmission ([19]). The result corresponds with regional studies indicating a significant frequency of dengue, underscoring the necessity of extensive preventative strategies ([20]).

Community involvement and public collaboration are essential for the efficacy of dengue control initiatives. Research in Negeri Sembilan, Malaysia, indicated that 38.8% of respondents were uncertain about the successful control of dengue, reflecting a deficiency in public trust in prevention strategies ([21]). If people continue to resist adopting preventive measures, dengue outbreaks are likely to endure, as collective community cooperation is essential for achieving lasting reductions in transmission (WHO, 2024). The research revealed a significant association between dengue awareness and the acceptance of diagnostic and preventive measures, including vaccination, Wolbachia-based mosquito control, and self-test dengue diagnostic kits ([22,23]).

Individuals with extensive knowledge of dengue fever are presumably more inclined to acknowledge the advantages of vaccine, early detection techniques, and vector control measures. This corresponds with studies on other infectious diseases, including human papillomavirus (HPV) vaccination and influenza prevention initiatives, wherein elevated public awareness is associated with enhanced vaccine acceptance and compliance with preventive measures ([22,23]). In the lack of a broadly accessible dengue vaccine or antiviral therapy, vector management is the principal approach for managing disease transmission. Studies indicate that extremely effective vector control programs can diminish herd immunity, requiring supplementary mosquito population reduction measures to avert recurrent epidemics ([24]).

The perceived benefit significantly predicts public views about dengue prevention methods. Research from Klang Valley indicates that residents are more likely to endorse vector control initiatives when they perceive substantial benefits from the intervention ([25]). This conclusion is substantiated by research on genetically modified mosquitoes and biodiesel applications, which revealed that perceived benefits had a greater impact on public acceptability than perceived risks ([26,27]). The impact of perceived advantages on public health decision-making extends beyond Malaysia. Research in Bandung, Indonesia, revealed robust popular endorsement for dengue immunization, with perceived advantages surpassing apprehensions regarding potential dangers ([29]). A study conducted in Kaohsiung City, Taiwan, revealed that public endorsement of aerosol pesticide applications and residential ovitraps resulted in a 60% decrease in dengue infections ([30]). These findings highlight the necessity of synchronizing dengue prevention initiatives with public emotion to enhance acceptance and efficacy.

This study underscores the necessity of incorporating diverse dengue control measures to enhance efficacy and public endorsement. Although frequently employed, fogging has limited short-term efficacy and reliance on public compliance, underscoring the necessity for additional strategies. The results highlight that perceived advantages and risk awareness substantially affect the public acceptability of vector control measures. Considering the significant association between public education and behavioral modification, subsequent studies should concentrate on formulating tailored awareness campaigns to enhance the acceptance of novel dengue prevention methods. Moreover, an in-depth examination of regional disparities in risk perception, socioeconomic variables, and behavioral responses would enhance the formulation of public health policies and intervention measures.

Dengue prevention programs that combine scientific innovation with community involvement can attain long-term decreases in transmission and morbidity, increasing their efficacy and sustainability.

REVIEWER 3

Reviewer #3: Aim of study :
1) This study does not explain in more detail the factors that influence a person's attitude and decision-making such as age, education, experience, social and cultural environment which is actually a matter of principle to describe the existing research population. Maybe this should be added to the data.

Response: Thank you for your comment. This is the refined version of the comment.

This study mainly examines the opinions of experts (scientists) and the public regarding dengue prevention techniques, particularly fogging and autocidal traps. In light of the research aims, the study excludes supplementary demographic criteria such as age, education, experience, or social and cultural context, as the primary focus is to examine attitudes based on stakeholder classification rather than individual demographic effects.

Although elements like as education and experience may affect individual opinions, our methodology focuses on contrasting viewpoints across the two primary stakeholder groups instead of analyzing differences within these categories based on demographic attributes. The exclusion of supplementary demographic elements was implemented to maintain a concentrated analysis that corresponds with the study's aims, so circumventing potential complexity could divert the research from its intended aim.

We recognize the significance of demographic factors in influencing decision-making processes. Future study should integrate these characteristics to enhance the understanding of how variables such as age, education, and cultural background influence stakeholders' opinions of dengue prevention efforts. We value your insightful contributions and invite continued dialogue regarding prospective avenues for exploration.

Reviewer #3: In study design : These samples represent people aged 18 years and above, and they have been stratified based on the target group. The stakeholders, consisting of scientists and the public, were divided into sub-groups based on the study site within the Klang Valley, Malaysia, specifically the districts and municipalities. The scientists were stratified by universities and departments, including ministries, institutes and health offices. The public was doing sampling in accordance with dengue hotspot regions mentioned on the i-dengue website provided by the Ministry of Health,Malaysia.

2) Can you more explain how many group you have been stratified ? There are no data for. The scientists and publics were stratified.

Response: Thank you for your comment. This is the refined version of the comment.

Thank you for your inquiry. This study categorized participants into two primary stakeholder groups: scientists (n = 212) and the public (n = 202). The scientists were categorized according to their professional affiliations, which included universities, academic departments, ministries, research institutes, and health agencies. The public group was categorized based on their geographical position inside dengue hotspot locations in the Klang Valley, as defined by the i-Dengue platform of the Ministry of Health, Malaysia.

However, the subgroup classification was not included in the final dataset; it was used primarily for organizational purposes when the survey was administered. The data collection and analysis relied exclusively on the overarching stakeholder classification (scientists or public), without additional stratification into particular institutions, districts, or localities. Although we recorded the locations during data entry, this information was not utilized as a variable for analysis, as our main objective was to evaluate replies at the stakeholder group level.

Consequently, the data lacks further subcategories beyond the two stakeholder groups, ensuring a clear and consistent assessment without introducing unintended classification criteria for analysis. We value your opinion and are willing to offer additional clarity if necessary.

Reviewer #3: In Acceptance of Dengue Alternative Techniques, they were provided with a scale of 7 response levels, ranging from "very strongly disagree" to "very strongly agree."

3) Please explain in detail the scale division of the 7 response levels of the limit in the selection of the answer option ?

Response: Thank you for your comment. This is the refined version of the comment.

This study adopted a 7-point Likert scale to evaluate the participants' attitudes and perspectives concerning fogging and autocidal traps as approaches for dengue prevention. The Likert scale offers a systematic method for gathering opinions, allowing respondents to express their degree of agreement or acceptance of a certain assertion. Frequently employed in social and health sciences research, this scale facilitates a more sophisticated assessment of attitudes than a basic binary response system (Likert, 1932; Boone & Boone, 2012).

Responses on the 7-point Likert scale are organized as follows: 1 (Strongly Disagree), 2 (Disagree), 3 (Somewhat Disagree), 4 (Neutral), 5 (Somewhat Agree), 6 (Agree), 7 (Strongly Agree). By using this method, researchers can examine subtle changes in attitudes instead of categorizing respondents into strict "agree/disagree" groups (DeVellis, 2017). In order to evaluate the average scores from Likert scale replies, the study divides the findings into three acceptance levels: low, moderate, and high. This classification aids in recognizing patterns in stakeholder perspectives about the two vector control techniques. According to prior studies in public health behavior (Petty et al., 1997; Dawes, 2008), the division adheres to these computed mean score ranges:

> Low Acceptance (1.00 - 3.00): Responses within this range signify strong disagreement, disagreement, or mild disagreement, indicating a low level of acceptance for the assessed approach or tactic. A mean score within this range indicates that respondents view the intervention as ineffective, superfluous, or adverse (Ajzen, 1991).
>
> Moderate Acceptance (3.01 - 5.00): This range encompasses neutral or partially agreeable responses, indicating ambivalence or conditional endorsement of the strategy. Individuals within this category may recognize the method's advantages, but they remain skeptical regarding its overall efficacy or practical implementation (Fishbein & Ajzen, 2010).
>
> High Acceptance (5.01 - 7.00): A mean score within this range signifies agreement to strong agreement, reflecting substantial acceptance and confidence in the procedure. Research indicates that elevated acceptance scores are associated with favorable behavioral intentions and readiness to embrace public health interventions (Bandura, 2004).

After data collection, the mean score for each statement is determined by respondents' choices using SPSS descriptive analysis. Upon obtaining the mean values, a three-tier categorization is employed to assess the level of acceptance of the intervention among respondents, classified as low, moderate, or high (Field, 2013). This approach guarantees a systematic, objective, and dependable categorization of attitudes, facilitating the assessment of perceptual disparities among stakeholder groups, including scientists and the public.

Implementing a 7-point scale and the classification into three acceptability levels enhances measurement precision relative to a 5-point scale, facilitating increased variation and diminishing central tendency bias (Brown, 2011). This method is especially useful in public health research because precise measurement of behavioral intentions and attitudes toward interventions is necessary to formulate effective policy recommendations (Rosenstock, 1974; WHO, 2024). This classification enables the study to delineate stakeholder views regarding fogging and autocidal traps, hence enhancing comprehension of the elements affecting public acceptance of vector management technologies. The outcomes obtained from this methodology can enhance public health communication strategies and intervention planning, ensuring that vector control initiatives correspond with stakeholder viewpoints and behavioral motivators.

Reviewer #3: The survey on attitudes towards alternate methods of preventing dengue consists of 24 items with Participants rate their agreement on a 7-point Likert scale, with 7 indicating strong agreement and 1 indicating strong disagreement.

4) Please explain in detail the scale division of the 7 response levels of the limit in the selection of the answer option ?

Response: thank you for your feedback. This is the refined version.

The explanation same as the above finding for the acceptance of the dengue alternative techniques.

Reviewer #3:

5) Perceived risk and benefit are not explained in terms of grouping and analysis

Response: Thank you for your feedback. This is the refined version of the feedback.

The analysis reveals that both principal stakeholder groups, scientists and the public, exhibit considerable interest in fogging techniques and autocidal traps, as illustrated in Table 1.3. Among the two dengue prevention approaches, autocidal traps garnered the greatest perceived benefit score, with scientists averaging 5.41 and the public 5.37. The general people regarded fogging as marginally less advantageous, with an average score of 5.29, whereas scientists assessed it at 5.20. A one-way multiple analysis of variance (MANOVA) was performed to evaluate the statistical differences in perception between the two stakeholder groups. The findings (Table 1.4) indicated a statistically significant disparity in the perceived advantages of fogging preventive measures ($F = 4.407$, $p < 0.05$; Pillai's Trace = 0.031) and autocidal traps ($F = 3.420$, $p < 0.05$; Pillai's Trace = 0.02). These results show that, at the multivariate level, stakeholder perceptions of the two dengue prevention strategies vary substantially. Nonetheless, when examined individually through univariate tests, the findings indicate no statistically significant difference in the average perception of benefits between fogging and autocidal traps. This indicates that although the stakeholder groups exhibit considerable variation in perception at the multivariate level, their general sentiments regarding the efficacy and advantages of both strategies remain largely consistent. This organized analysis of the findings offers an in-depth comprehension of stakeholder perceptions, facilitating a more distinct comparison of attitudes between scientists and the public regarding dengue prevention efforts.

Reviewer #3:

6) In the discussion it is not explained in more detail that knowledge of fogging and autocidal traps is greatly influenced by knowledge and experience in taking attitudes. Knowledge of the importance of environmental balance as an ecological place where disease vectors live will determine a person's choice to make decisions in preventive measures against vector-borne diseases so that environmental control becomes an important thing to be raised as disease prevention in addition to treatment.

Response: Thank you for your feedback. This is the revised and refined version of the feedback you provided.

Knowledge and experience profoundly affect attitudes and behaviors toward fogging and autocidal traps, as both techniques necessitate comprehension of their function in preserving

ecological equilibrium and managing disease vectors. Research indicates that increased awareness of dengue prevention is associated with improved attitudes and practices. Lugova and Wallis (2016) assert that comprehensive knowledge and positive attitudes regarding dengue substantially improve preventive measures in university environments, especially among educated individuals. Corresponding to this, Ajibola et al. (2018) point out that people's decisions to use preventive measures like fogging and autocidal traps are influenced by their understanding of Aedes mosquito behavior as well as socioenvironmental factors.

The ecological significance of sustaining a balanced ecosystem as a vector control technique corresponds with evidence highlighting the influence of environmental factors such as urbanization, water storage methods, and waste management on mosquito proliferation (Ajibola et al., 2018). Lugova and Wallis (2016) indicate that educational campaigns addressing knowledge deficiencies on mosquito breeding habits and diurnal biting behaviors can substantially enhance the efficacy of fogging and autocidal trap initiatives.

The cross-sectional study by Zamri et al. (2020) establishes a significant correlation between dengue-related knowledge and favorable attitudes toward preventive measures, indicating that enhancing awareness can improve community engagement. In addition to technical effectiveness, public awareness of the ecological and health benefits of fogging and autocidal traps is crucial for their successful adoption (Ajibola et al., 2018; Lugova and Wallis, 2016).

The synthesis of these data emphasizes the necessity of combining information distribution with practical demonstrations to enhance public adoption of advanced vector control methods. Future tactics must prioritize community-centered educational initiatives to rectify misconceptions and foster the adoption of novel dengue prevention techniques.

Reviewer #3:

7) The conclusion of the results should further explain the purpose of the study

Response: thank you for the feedback. This is the refined version of the feedback.

This study emphasizes the essential influence of public perception on the implementation and efficacy of dengue prevention efforts. Although fumigation is still broadly endorsed, apprehensions surrounding pesticide resistance, health hazards, and environmental contamination underscore the necessity for alternative and supplementary approaches, including autocidal traps and biological control methods. Research reveals that stakeholder perceptions of advantages and hazards substantially affect acceptance rates, with increased public endorsement when a method's efficacy is well established. The efficacy of dengue prevention efforts depends on public acceptance and adherence. Both conventional fogging and innovative autocidal traps necessitate substantial acceptance and favorable attitudes for effective use. When individuals acknowledge the advantages of preventative actions, such as

eradicating mosquito breeding areas and implementing protective methods, they are more inclined to adopt and maintain these activities. This study investigated stakeholder perceptions of fogging and autocidal traps, demonstrating that perceived advantages and hazards substantially influence adoption and effectiveness. The results underscore the necessity of a comprehensive dengue prevention strategy that incorporates several control measures to improve overall efficacy and reduce disease burden. A comprehensive, evidence-based strategy is crucial for dengue's sustainable and enduring prevention. Dengue prevention efforts can be greatly improved by combining public health measures, personal safety measures, and community-led actions. The amalgamation of scientific progress, public awareness campaigns, and environmental stewardship is essential for enhancing the sustainability and effectiveness of vector control methods. The efficacy of these treatments relies on cultivating favorable public impressions while mitigating concerns regarding safety, effectiveness, and accessibility. The success of dengue control initiatives depends on raising public awareness, fostering confidence in emerging technology, and promoting community involvement. This study highlights the importance of evidence-based public health strategies that prioritize community engagement and integrated approaches. Enhancing public comprehension and encouraging active engagement will foster a more resilient and proactive society, so effectively diminishing dengue transmission and protecting public health. Subsequent studies ought to investigate the socioeconomic and behavioral determinants affecting the uptake of innovative vector control methods, establishing a basis for evidence-based public health policies. By combining new scientific discoveries with active community participation, dengue prevention efforts can be more successful and last longer, reducing the impact of dengue fever around the world.
