# Supplementary material for: Stakeholder acceptance and attitudes toward dengue prevention: A study on fogging and autocidal traps techniques in Malaysia
Source: PLoS One. 2025 Jul 16;20(7):e0323061. doi: 10.1371/journal.pone.0323061 (PMC12266440; doi:10.1371/journal.pone.0323061)
Supplement: Attachment — Submitted filename: Response to Reviewers.docx [file pone.0323061.s003.docx]

**Comments to the Author**

1. If the authors have adequately addressed your comments raised in a previous round of review and you feel that this manuscript is now acceptable for publication, you may indicate that here to bypass the “Comments to the Author” section, enter your conflict of interest statement in the “Confidential to Editor” section, and submit your "Accept" recommendation.

Reviewer #1: All comments have been addressed

Reviewer #2: All comments have been addressed

Reviewer #4: (No Response)

2. Is the manuscript technically sound, and do the data support the conclusions?

The manuscript must describe a technically sound piece of scientific research with data that supports the conclusions. Experiments must have been conducted rigorously, with appropriate controls, replication, and sample sizes. The conclusions must be drawn appropriately based on the data presented.

Reviewer #1: Yes

Reviewer #2: Yes

Reviewer #4: Partly

3. Has the statistical analysis been performed appropriately and rigorously?

Reviewer #1: Yes

Reviewer #2: Yes

Reviewer #4: No

4. Have the authors made all data underlying the findings in their manuscript fully available?

The [PLOS Data policy](http://track.editorialmanager.com/CL0/http:%2F%2Fwww.plosone.org%2Fstatic%2Fpolicies.action%23sharing/1/010f01969158809e-5442f0cd-3cc4-4436-9243-78f909fc9ef5-000000/vZdK_zWOBLHXdlxZTaDduifNU44pj87qGJTcfihV2Ho=209) requires authors to make all data underlying the findings described in their manuscript fully available without restriction, with rare exception (please refer to the Data Availability Statement in the manuscript PDF file). The data should be provided as part of the manuscript or its supporting information, or deposited to a public repository. For example, in addition to summary statistics, the data points behind means, medians and variance measures should be available. If there are restrictions on publicly sharing data—e.g. participant privacy or use of data from a third party—those must be specified.

Reviewer #1: Yes

Reviewer #2: Yes

Reviewer #4: No

5. Is the manuscript presented in an intelligible fashion and written in standard English?

PLOS ONE does not copyedit accepted manuscripts, so the language in submitted articles must be clear, correct, and unambiguous. Any typographical or grammatical errors should be corrected at revision, so please note any specific errors here.

Reviewer #1: Yes

Reviewer #2: Yes

Reviewer #4: Yes

6. Review Comments to the Author

Please use the space provided to explain your answers to the questions above. You may also include additional comments for the author, including concerns about dual publication, research ethics, or publication ethics. (Please upload your review as an attachment if it exceeds 20,000 characters)

Reviewer #1: The authors of the scientific work have responded with rigor and clarity to the questions and recommendations of the manuscript. I consider that this is a publication that meets the criteria of the journal. Its publication contributes with strong evidence to guide public health decisions for the sustainable prevention of diseases transmitted by Aedes spp. mosquitoes.

Reviewer #2: (No Response)

Reviewer #4: This is a good descriptive presentation. However, the inferential statistical approach is not necessarily robust as claimed and needs justification. Also, further detail should be considered as follows:

Please justify the MANOVA analysis since this is likert type data. Why not a non parametric type of analysis or other approach for this kind of data? Was the data checked for normality?
An expanded statistical analysis section is needed for all primary and secondary analysis endpoints need an expanded statistical analysis section.

Also the stakeholder groups are not randomized which calls into question the MANOVA approach. Was a propensity matching considered for the two groups since formal comparative statistical tests are being performed on a non randomized sample. The sample size looks large enough to tabulate the demographics, if any, were selected in the data collection.

The amended text now contains an enlarged statistical analysis section that outlines all checked assumptions, reasons for parametric tests, and the rationale for the selection of MANOVA.

*We recognize the reviewer's apprehension about applying MANOVA to Likert-type data. Although individual Likert items are ordinal, our analysis utilized composite mean scores (e.g., perceived benefit, perceived danger, and acceptance), each generated from numerous Likert-scale items. This methodology is extensively endorsed in the literature, wherein aggregated scores from several items are regarded as resembling interval-level data and are frequently examined using parametric methods when specific assumptions are met (Norman, 2010; Harpe, 2015).*

*Prior to conducting the MANOVA, we checked for the following assumptions:*

*- Normality: Skewness and kurtosis values for the composite variables were within acceptable ranges (±2), and visual inspection of Q-Q plots confirmed approximate normal distribution.*

*- Homogeneity of variances and covariances: Assessed using Levene’s Test and Box’s M Test respectively, with results supporting the assumption.*

*- Multicollinearity: Pearson correlation values between dependent variables were moderate and did not exceed 0.8, confirming that the constructs were related but distinct.*

*- Equality of sample sizes: The stakeholder groups were nearly equal (Scientist = 212; Public = 203), supporting balanced group comparison in MANOVA.*

*Pillai’s Trace was chosen as the test statistic because it is resilient to breaches of MANOVA assumptions. Statistically significant variations among groups were identified for many dependent variables (e.g., perceived risk for autocidal trap, p = 0.006), validating the sensitivity and suitability of MANOVA in this setting.*

*Besides, we recognize that the stakeholder groups were not randomized because this study was observational and exploratory. The groups (scientists and the public) were delineated according to actual stakeholder roles rather than being allocated through experimental design. The objective was not to demonstrate causal relationships but to compare perceptions among existing positions.*

*Although propensity score matching effectively reduces bias in quasi-experimental comparisons, it was not utilized in this instance due to the equitable group sizes and the study's emphasis on stakeholder disparities rather than intervention effects. We concur that demographic confounding constitutes a significant constraint; thus, we have incorporated a comprehensive tabulation of demographic data and highlighted this limitation in the discussion.*

• Norman, G. (2010). *Likert scales, levels of measurement and the “laws” of statistics*. Advances in Health Sciences Education, 15(5), 625–632.

• Harpe, S. E. (2015). *How to analyze Likert and other rating scale data*. Currents in Pharmacy Teaching and Learning, 7(6), 836–850.

The Discussion section needs to list the advantages and limitations encountered in this research.

*However, this study has certain analytical and methodological benefits. Comparisons between the public and scientific groups were made methodically using validated categories and composite mean scores to assess stakeholder views of fogging and autocidal traps. The application of MANOVA enabled the simultaneous assessment of many dependent variables, improving the effectiveness and robustness of group comparisons. The sample size (n=415) provided considerable statistical power to detect significant differences, while segmentation by stakeholder group ensured thorough representation of public and scientific perspectives.*

*Nonetheless, specific limitations must be acknowledged. The study employed non-randomized groupings, limiting the ability to reduce selection bias in stakeholder comparisons. Although the statistical assumptions for MANOVA have been satisfied through testing, the interpretation of Likert-scale data may still provide issues, as it is regarded as an interval for parametric analysis. Moreover, due to its cross-sectional design, the findings reflect perspectives at a specific time and do not account for changes in acceptance or awareness over time. Despite these limitations, the findings provide a substantial overview of stakeholder perspectives and may inform the development of future public health communication and engagement methods.*

7. PLOS authors have the option to publish the peer review history of their article ([what does this mean?](https://track.editorialmanager.com/CL0/https:%2F%2Fjournals.plos.org%2Fplosone%2Fs%2Feditorial-and-peer-review-process%23loc-peer-review-history/1/010f01969158809e-5442f0cd-3cc4-4436-9243-78f909fc9ef5-000000/QYu4lye4d_Vt7NvujzwYY1F9JLqn0EUc0-Q3r53wH20=209)). If published, this will include your full peer review and any attached files.


If you choose “no”, your identity will remain anonymous but your review may still be made public.


**Do you want your identity to be public for this peer review?** For information about this choice, including consent withdrawal, please see our [Privacy Policy](https://track.editorialmanager.com/CL0/https:%2F%2Fwww.plos.org%2Fprivacy-policy/1/010f01969158809e-5442f0cd-3cc4-4436-9243-78f909fc9ef5-000000/_5pD-v_ldqH40-zN_zy66FnEQTm_GYtZQS5MRVyHhp8=209).

Reviewer #1: **Yes:** Ángel Betanzos Reyes

Reviewer #2: No

Reviewer #4: No
